# Relationship between leukocyte count and renal function in patients with lupus nephritis: a retrospective study of Chinese adults

**DOI:** 10.1177/03000605241305433

**Published:** 2024-12-24

**Authors:** Jianyu Chen, Rong Cao, Jianying Guo, Yang Liu, Haofei Hu, Qijun Wan

**Affiliations:** 1 Department of Nephrology, the First Affiliated Hospital of Shenzhen University, Shenzhen, China; 2 Department of Nephrology, the Second People’s Hospital of Shenzhen, Shenzhen, China

**Keywords:** Lupus nephritis, leukocyte, renal function, estimated glomerular filtration rate, microthrombus, 24-hour proteinuria

## Abstract

**Objective:**

To explore the relationship between leukocyte count and renal function in patients with lupus nephritis (LN).

**Methods:**

We performed a retrospective cross-sectional study of 100 patients with LN admitted to the Department of Nephrology between 1 January 2015 and 30 December 2023. Based on tertile of leukocyte count, they were allocated to Low, Medium, or High groups. The demographic, clinical, and pathologic characteristics of the groups were compared and the relationship between leukocyte count and renal function was analyzed using multiple linear regression analysis.

**Results:**

After adjustment for age, albumin, 24-hour proteinuria, serum complement factor 3, hemoglobin, blood pressure, systemic lupus erythematosus disease activity index score, and histologic parameters, a linear relationship was identified between leukocyte count and renal function (regression coefficient −2.852; 95% confidence interval: −5.161, −0.543). Therefore, for every 1 × 10^9^/L increase in leukocyte count, the estimated glomerular filtration rate would decrease by 2.85 mL/minute.

**Conclusion:**

There is an independent linear relationship between leukocyte count and renal function. Leukocyte recruitment into the kidney is a critical step in the progression of LN, and as the leukocyte count increases, renal function gradually declines. Leukocyte count may represent a sensitive biomarker of the progression and prognosis of LN.

## Introduction

Systemic lupus erythematosus (SLE) is a systemic autoimmune disease characterized by the deposition of circulating immune complexes, which are formed by the aggregation of autoantibodies and antigens and cause inflammation by promoting the tissue infiltration of various types of leukocytes.^1^

It is estimated that 10% of patients with SLE progress to end-stage renal disease (ESRD) within 5 years of onset, secondary to the development of lupus nephritis (LN). The overall prevalence of LN is currently >50% in patients with SLE, and the prevalences in Asian and African people are 55% and 51%, respectively.^2^ Overt LN presents clinically on a spectrum from asymptomatic microscopic hematuria to renal failure, and even in the presence of no clinical or laboratory signs of renal involvement, some patients with silent LN may demonstrate pathologic evidence of renal involvement on renal biopsy.^3^ Indeed, an early study by Wakasugi *et al*.^4^ showed that 73% of patients with SLE but no clinical signs of renal involvement have silent LN. Circulating immune complexes and subsequent complement activation cause injury to renal cells, leading to the release of proinflammatory factors and subsequent leukocyte infiltration of the glomeruli, renal tubulointerstitium, and perivascular areas, which promotes renal inflammation and damage.^5^ In this way, the recruitment of leukocytes to the kidney plays a crucial role in the progression of LN.^6^ Previous research has demonstrated that markers of inflammation, such as the leukocyte count and the circulating C-reactive protein and interleukin-6 concentrations, correlate with renal function and serve as predictors of future renal deterioration and the risk of developing chronic kidney disease.^7^ Nonetheless, the association between leukocyte levels and renal function in Chinese patients with lupus nephritis has not been documented. In the present study, we aimed to characterize the relationship between leukocyte count and renal function in patients with LN.

## Materials and methods

### Study design and patients

We performed a retrospective cross-sectional study that was approved by the Medical Ethics Committee of Shenzhen Second People’s Hospital (approval number: 20211108007). It was performed according to the Declaration of Helsinki of 1975, as revised in 2013,^8^ and is reported in accordance with the STROBE guidelines.^8^ We recruited consecutive patients with LN who were admitted to the Second People’s Hospital of Shenzhen between January 2015 and December 2023, encoding their identity using untraceable codes to protect their privacy. Data were collected from the hospital electronic medical records. Verbal informed consent was obtained from each of the participants.

### Eligibility criteria

To be eligible for inclusion, the patients had to satisfy the 2010 revised classification criteria for SLE, as established by the American College of Rheumatology,^9^ and have a confirmed diagnosis of LN made by histologic examination of a renal biopsy.

### Exclusion criteria

The exclusion criteria were concurrent tumor, severe cardiovascular disease, or severe infection, and an incomplete set of clinical and pathologic data. The patients were allocated to three groups according to their leukocyte count: tertile (T)1, ≤4.38 (Low), T2, 4.39 to 6.30 (Moderate), and T3 >6.30 × 10^9^/L (High).

### Sociodemographic and clinical assessments

After enrollment, the demographic, clinical, and pathologic data for each patient were systematically collected. The demographic data included the age, sex, systolic blood pressure (SBP), diastolic blood pressure (DBP), and body mass index (BMI) of the patients; and the pathologic parameters included the presence or absence of renal tubular atrophy, the total proportion of glomerular crescents, and the presence or absence of endotheliosis. Hypertension was defined using an SBP of ≥140 mmHg, a DBP of ≥90 mmHg, a clinical diagnosis of hypertension, or contemporaneous use of antihypertensive medication.^10^

### Laboratory assessments

The medical histories and laboratory test results of the patients were recorded, and blood and urine samples were obtained for the measurement of clinically relevant parameters. Venous blood samples were drawn from an antecubital vein following a 12-hour fast prior to renal biopsy, and standard laboratory methods were used to measure the circulating parameters, including the serum creatinine (Scr), hemoglobin (Hb), serum albumin (ALB), and complement factor (C)3 concentrations. The patients collected all the urine voided over a 24-hour period to quantify protein excretion. All the baseline data were obtained prior to the renal biopsy procedure. Renal tissue samples were dispatched to Kingmed Diagnostics, Guangzhou, China, where they underwent light microscopic, immunofluorescence, and electron microscopic evaluations. Pathologic assessments were conducted by at least two expert pathologists, who independently reviewed the samples multiple times and were blinded to the patients’ clinical outcomes.

### Assessment of disease activity

Lupus disease activity was evaluated using the systemic lupus erythematosus disease activity index (SLEDAI) score.^11^

### Key variable of interest

The primary independent variable of interest was the leukocyte count at baseline, which was analyzed as a continuous variable.

### Primary outcome

The primary outcome was the estimated glomerular filtration rate (eGFR), which was calculated using the Modification of Diet in Renal Disease equation as follows: eGFR (mL/minute/1.73 m^2^) = 186.3 × [Scr (µmol/L) × 0.0113] − 1.154 × age − 0.203 (×0.742 for women).^12^

### Covariates

The following variables were regarded as covariates: (1) continuous variables: age, ALB, 24-hour proteinuria, serum C3, Hb, SBP, DBP, eGFR, SLEDAI, and the proportion of glomerular crescents; and (2) categorical variables: sex, nuclear fragmentation, mesangial cell and matrix proliferation, microthrombus, endotheliosis, and renal tubule atrophy. Data were collected under standardized conditions and biochemical parameters were measured according to standard procedures using standard automated analyzers.

### Statistical analysis

We used R (R Foundation for Statistical Computing, Vienna, Austria; http://www.R-project.org) and Empower-Stats (X&Y Solutions, Inc., Boston, MA, USA; http://www.empowerstats.com) for all the analyses. Statistical significance was accepted when *P* < 0.05 (two-sided). Normally distributed continuous datasets are presented as mean ± standard deviation and were compared using one-way ANOVA. Non-normally distributed continuous datasets are expressed as median and interquartile range and were compared using the Kruskal–Wallis test. Categorical datasets are presented as frequency (percentage) and the chi-square test was used for intergroup comparisons. Univariate and multivariate linear regression analyses were conducted to explore the relationship between leukocyte count and renal function, and a curve-fitting analysis was conducted to determine the nature of the relationship between these parameters.

## Results

### General clinical data

**Figure 1.**
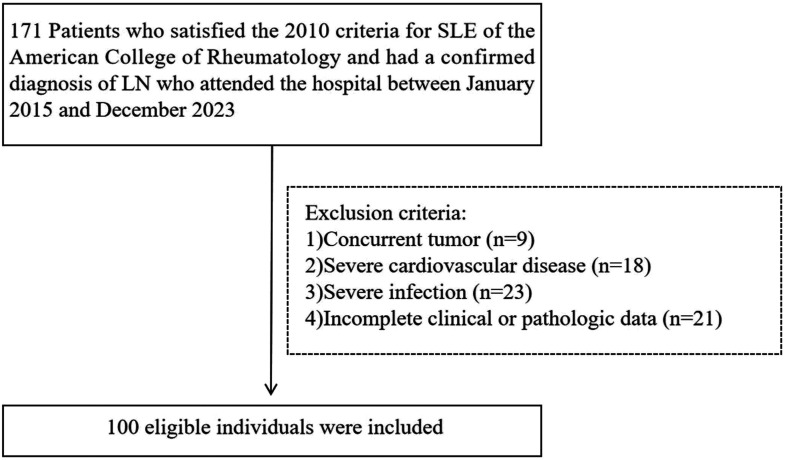
Study design and inclusion of participants. SLE, systemic lupus erythematosus; LN, lupus nephrosis.

We initially enrolled 171 individuals, of whom 71 were excluded. Therefore, we analyzed data for 100 patients (Figure 1), who comprised 6 men and 94 women. The numbers of patients in the Low, Moderate, and High leukocyte count groups were 33, 33, and 34, respectively. Table 1 shows the baseline characteristics of the patients. There were no significant differences in the sex, age, or other demographic data between the three groups.

**Table 1.** Baseline characteristics of the participants.

Variable	Leukocyte count, ×10^9^/L			*P*-value
	Q1 (≤4.38)	T2 (4.38 to 6.29)	T3 (>6.30)	
Participants (n)	33	33	34	
Age (years)	32.82 ± 7.04	34.88 ± 12.32	32.56 ± 11.71	0.623
ALB (μmol/L)	429.6 ± 151.8	420.3 ± 181.3	387.6 ± 125.6	0.508
24-hour proteinuria (g/24 hours)	2.98 ± 3.02	2.90 ± 3.35	2.65 ± 2.35	0.894
Serum C3 (mg/dL)	0.54 ± 0.30	0.66 ± 0.27	0.62 ± 0.26	0.226
Hb (g/L)	101.55 ± 19.69	108.02 ± 24.54	101.36 ± 22.66	0.389
SBP (mmHg)	122.21 ± 15.47	128.06 ± 20.49	133.09 ± 23.90	0.095
DBP (mmHg)	78.39 ± 10.37	81.03 ± 12.61	83.47 ± 13.18	0.235
eGFR (mL/minute/1.73 m^2^)	100.04 ± 31.65	89.33 ± 39.92	77.51 ± 47.16	0.076
SLEDAI	14.21 ± 4.91	14.12 ± 5.70	14.24 ± 6.96	0.997
Proportion of glomerular crescents	7.23 ± 10.40	12.88 ± 19.28	20.02 ± 25.27	0.029
Nuclear fragmentation, n (%)				0.781
absent	11 (33.33)	11 (33.33)	9 (26.47)	
present	22 (66.67)	22 (66.67)	25 (73.53)	
Mesangial cell and matrix proliferation, n (%)				0.509
absent	0 (0.00)	0 (0.00)	2 (5.88)	
Grade 1	9 (27.27)	11 (33.33)	12 (35.29)	
Grade 2	14 (42.42)	15 (45.45)	13 (38.24)	
Grade 3	10 (30.30)	7 (21.21)	7 (20.59)	
Microthrombus, n (%)				0.609
absent	21 (63.64)	21 (63.64)	25 (73.53)	
present	12 (36.36)	12 (36.36)	9 (26.47)	
Endotheliosis, n (%)				0.498
absent	5 (15.15)	8 (24.24)	9 (26.47)	
present	28 (84.85)	25 (75.76)	25 (73.53)	
Renal tubular atrophy, n (%)				0.503
absent	19 (57.58)	20 (60.61)	16 (47.06)	
present	14 (42.42)	13 (39.39)	18 (52.94)	

Values are n (%) or mean ± standard deviation. The groups were compared using one-way ANOVA. eGFR, estimated glomerular filtration rate; C3, complement factor 3; SBP, systolic blood pressure; DBP, diastolic blood pressure; ALB, albumin; Hb, hemoglobin; SLEDAI, systemic lupus erythematosus disease activity index.

### Results of the univariate analysis

Univariate linear regression analysis was conducted to identify factors influencing the renal function of patients with LN. Univariate analysis revealed that age; the presence of microthrombi, nuclear fragmentation, endothelial hyperplasia, and tubular atrophy; the proportion of glomerular crescents; and SBP and DBP negatively correlated with renal function, indicating that these parameters are risk factors for impaired renal function in patients with LN (Table 2). In addition, Hb positively correlated with renal function, suggesting that it was a protective factor.

**Table 2.** Results of the univariate analysis.

Variable	Statistic	HR (95% CI)	*P*-value
Age (years)	33.41 + 10.58	−0.96 (−1.70,−0.21)	0.013
Nuclear fragmentation, n (%)			
absent	31 (31.00)	ref	
present	69 (69.00)	−39.01 (−54.59,−23.44)	<0.0001
Mesangial cell and matrix proliferation, n (%)			
absent	2 (2.00)	ref	
1	32 (32.00)	59.99 (7.38, 112.60)	0.028
2	42 (42.00)	37.19 (−15.05, 89.43)	0.166
3	24 (24.00)	11.89 (−41.23, 65.01)	0.662
Microthrombus, n (%)			
absent	67 (67.00)	ref	
present	33 (33.00)	−25.05 (−41.42, −8.69)	0.003
ALB (g/L)	27.40 + 10.22	0.23 (−0.56, 1.02)	0.571
24-hour proteinuria (g/24 hours)	2.84 + 2.91	−1.67 (−4.43, 1.10)	0.240
Endotheliosis, n (%)			
absent	22 (22.00)	ref	
present	78 (78.00)	−36.76 (−54.75, −18.77)	0.0001
Serum C3 (mg/dL)	0.61 + 0.28	12.32 (−17.15, 41.78)	0.415
Hb (g/L)	103.62 + 22.38	0.88 (0.57, 1.20)	<0.0001
SBP (mmHg)	127.84 + 20.58	−1.03 (−1.37, −0.70)	<0.0001
DBP (mmHg)	80.99 + 12.18	−0.87 (−1.51, −0.23)	0.009
SLEDAI	14.19 + 5.87	−1.09 (−2.45, 0.27)	0.121
Proportion of glomerular crescents	13.44 + 19.90	−1.00 (−1.35, −0.64)	<0.0001
Renal tubular atrophy, n (%)			
absent	55 (55.00)	Ref	
present	45 (45.00)	−24.01 (−39.46, −8.56)	0.003

Values are n (%) or mean ± standard deviation. The groups were compared using one-way ANOVA. C3, complement factor 3; ALB, albumin; Hb, hemoglobin; SBP, systolic blood pressure; DBP, diastolic blood pressure; SLEDAI, systemic lupus erythematosus disease activity index.

### Results of the multiple linear regression analysis

Multivariate linear regression models were next created to further evaluate the relationship between leukocyte count and renal function. The results of the unadjusted and adjusted models are shown in Table 3. In the unadjusted model, there was a negative correlation between leukocyte count and renal function (β = −3.346, 95% confidence interval [CI]: −5.736 to −0.956, *P* < 0.05). In the model that was adjusted for age and hypertension, the relationship identified was similar (β = −2.667, 95% CI: −5.037 to −0.298, *P* < 0.05), and in the model that was adjusted for age, hypertension, nuclear fragmentation, mesangial cell and matrix proliferation, microthrombi, ALB, serum C3, 24-hour proteinuria, endothelial hyperplasia and Hb, the relationship remained (β = −2.852, 95% CI: −5.161 to −0.543, *P* < 0.05). As a sensitivity analysis, we treated the leukocyte count as a categorical variable (tertiles) and repeated the analysis, obtaining a similar relationship (*P* = 0.04). Thus, we identified a robust negative relationship between leukocyte count and renal function.

**Table 3.** Relationships between leukocyte count and renal function identified using the various multivariable regression models

	Unadjusted model	Minimally-adjusted model	Fully-adjusted model
	HR (95% CI); *P*	HR (95% CI); *P*	HR (95% CI); *P*
Leukocyte count	−3.35 (−5.74, −0.96); 0.007	−2.67 (−5.04, −0.30); 0.030	−2.85 (−5.16, −0.54); 0.018
Leukocyte count groups			
T1 (≤4.38 × 10^9^/L)	Ref	Ref	Ref
T2 (4.39 to 6.30 × 10^9^/L)	−10.71 (−30.08, 8.67); 0.282	−8.46 (−26.72, 9.78); 0.366	−14.65 (−30.67, 1.38); 0.077
T3 (>6.30 × 10^9^/L)	−22.53 (−41.77,−3.30); 0.024	−16.75 (−35.32, 1.82); 0.080	−18.29 (−35.54, −1.05); 0.040

Unadjusted model: we did not adjust for covariates. Minimally-adjusted model: we adjusted for age and hypertension. Fully-adjusted model: we adjusted for age, hypertension, nuclear fragmentation, mesangial cell and matrix proliferation, microthrombi, albumin, serum C3, 24-hour proteinuria, endothelial hyperplasia, and hemoglobin. HR, hazard ratio; CI, confidence interval; Ref, reference.

### Curve fitting

**Figure 2.**
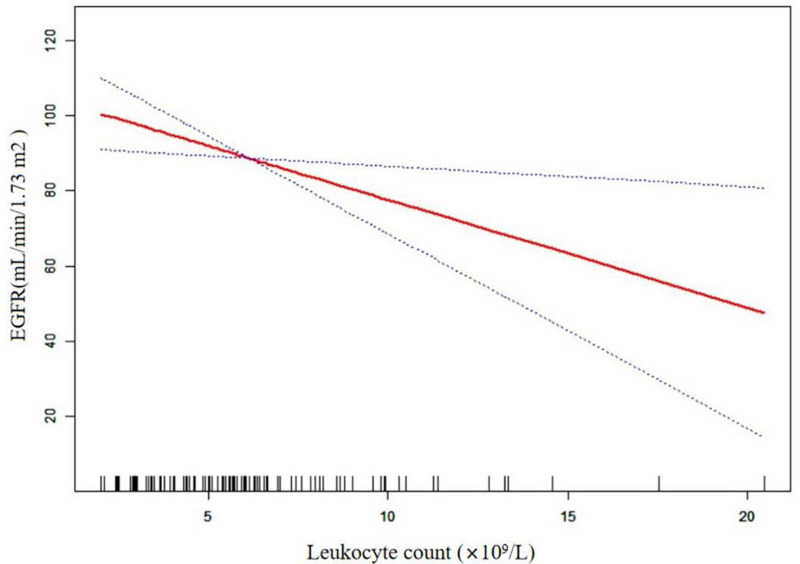
Results of the curve fitting analysis. eGFR, estimated glomerular filtration.

We further explored the relationship between leukocyte count and renal function by means of a curve fitting analysis. This showed that the relationship between these parameters was linear, rather than curved, after adjustment for age, hypertension, nuclear fragmentation, mesangial cell and matrix proliferation, microthrombi, ALB, serum C3, 24-hour proteinuria, endothelial hyperplasia, and Hb (Figure 2). Thus, as the leukocyte count increases, renal function gradually declines, without a saturation effect.

## Discussion

In the present cross-sectional study, we have evaluated the relationship between the leukocyte count and renal function of Chinese patients who had been diagnosed with LN. Using multivariate linear regression, we identified a significant linear relationship between the two parameters, and this relationship remained in the fully adjusted model (adjusted for age, hypertension, nuclear fragmentation, mesangial cell and matrix proliferation, microthrombi, ALB, serum C3, 24-hour proteinuria, endothelial hyperplasia, and Hb), with a regression coefficient (β-value) of −2.852 (95% CI: −5.161, −0.543; *P* = 0.01759). This indicates that for each increase of 1 × 10^9^/L in the leukocyte count, the eGFR of the patient would decrease by 2.85 mL/minute. We further explored the relationship by means of curve fitting analysis, and found that it was linear, rather than curved (Figure 2). Thus, as the leukocyte count increases, renal function gradually declines, without a saturation effect. These findings suggest that the leukocyte count may represent a useful means of assessing renal function in Chinese people with LN.

SLE is an autoimmune disease that can be life-threatening and is underpinned by a complex immune pathogenesis that is not completely understood. The current criteria for remission are based on the results of laboratory testing for parameters such as Scr and proteinuria, but these tests are not able to differentiate between active disease and existing organ damage. Furthermore, multiple studies have shown that such tests have low sensitivity for the prediction of disease outcomes.^13–15^

Hematologic complications are common in patients with SLE and include low leukocyte and lymphocyte, and platelet counts, autoimmune hemolytic anemia, and thrombocytopenic purpura.^16^ These hematologic manifestations are closely associated with disease activity and contribute to a poor prognosis. The causes of the hematologic involvement in SLE are complex, but LN is typically characterized by a low leukocyte count. However, as the incidence of SLE has increased, a high leukocyte count has been identified in an increasing number of patients, but its effect on disease progression remains unclear. A high leukocyte count often develops during the stable phase of SLE, and this may be closely associated with the occurrence of inflammatory reactions.^17,18^ Patients with SLE produce a large number of autoantibodies, including anti-leukocyte and anti-platelet antibodies, which bind to the corresponding antigens on leukocyte and platelet membranes, activate the complement system, and cause cell lysis. This phenomenon generally occurs during the active phase of lupus. When multiple organs, including the kidneys, are affected, a large number of cells are destroyed, and therefore approximately 40% of patients with SLE experience leukopenia.^19^ In addition, anti-neutrophil cytoplasmic antibodies may be present. Thus, the leukocyte count may provide information regarding disease progression and the prognosis of the patient. However, the peripheral leukocyte count has been reported not to be an independent predictor of systemic infection and has been shown not to be an accurate means of diagnosing systemic infection, although it does reflect the severity of infection.^20^

Recent studies have shown that granulocytes, including neutrophils, basophils, and eosinophils, are key players in the development of LN erythematosus because they drive abnormal autoimmune responses, which cause tissue damage.^6^ Various types of leukocyte migrate into the kidneys of patients with lupus nephritis following interactions between chemokines and chemokine receptors, which, together with the activation of renal parenchymal cells by proinflammatory chemokines, leads to acute or chronic LN. Chemokines are small cytokines that primarily attract specific leukocyte types in normal individuals and in those with various diseases. The recruitment of leukocytes to inflamed kidneys is a crucial step in the progression of LN.^21^

Liang *et al.* identified a negative correlation between the circulating basophil count and the renal activity of LN by analyzing data obtained from 159 patients who had received a diagnosis on the basis of renal biopsy. This finding suggests that the basophil count might be a predictor of greater renal activity of LN and relatively severe renal damage.^22^ A potential explanation for this correlation is the involvement of basophils in the pathogenesis of LN through the production of T-helper 2-derived proinflammatory cytokines, such as interleukin-4 and thymic stromal lymphopoietin. These cytokines may exacerbate renal dysfunction and stimulate T and B cells to produce autoantibodies.^23^ Han *et al*. demonstrated that the neutrophil-to-lymphocyte ratio positively correlates with lupus activity,^24^ and this is consistent with the findings of previous studies.^25–27^ Neutrophils are the most abundant type of leukocyte in humans, and patients with SLE show abnormal neutrophil phenotypes and functions, such as greater aggregation and impaired phagocytosis.^6^ In addition, there are increases in the numbers of apoptotic and secondary necrotic neutrophils as disease activity increases.^28^ Zhao *et al*. reported that the standard deviation of lymphocyte volume is associated with SLE disease activity. Furthermore, the combination of the standard deviation of lymphocyte volume, red blood cell count, and lymphocyte percentage has been shown to be a useful predictor of SLE activity.^29^

Patients with SLE require an early and accurate diagnosis, close monitoring of disease activity, and the timely provision of accurate information to ensure effective management. Clinicians need to be able to control inflammation and tissue damage in their patients because inflammation is a crucial factor in the recurrent exacerbation of SLE.

Leukocytes are closely associated with the progression of LN, and the present study has demonstrated a robust linear correlation between leukocyte count and kidney function. Therefore, it might have potential for the assessment of renal function in individuals with LN. However, it is important to acknowledge certain limitations of the study. First, it was a single-center retrospective study that may have been subject to bias owing to the restrictions to the dataset. Consequently, further larger, multicenter studies should be performed to validate the findings. Second, because it was a cross-sectional study, it is not possible to infer a causal link between the exposure and outcome.

## Conclusion

We have identified a robust linear relationship between leukocyte count and renal function in patients with LN, implying that the higher a patient’s leukocyte count is, the worse is their renal function. The leukocyte count may represent a useful predictor of the prognosis of LN.
